# *N*-Benzyl­thieno[3,2-*d*]pyrimidin-4-amine

**DOI:** 10.1107/S1600536813009537

**Published:** 2013-04-13

**Authors:** Pavel Štarha, Zdeněk Trávníček

**Affiliations:** a Department of Inorganic Chemistry, Faculty of Science, Palacký University, 17. listopadu 12, CZ-771 46 Olomouc, Czech Republic

## Abstract

The title compound, C_13_H_11_N_3_S, crystallizes with two independent mol­ecules in the asymmetric unit. The two mol­ecules are geometrically very similar and differ mainly in a spatial orientation of the benzene and thieno[3,2-*d*]pyrimidine ring systems [dihedral angles = 69.49 (4) and 79.05 (3)°]. The nine-membered thieno[3,2-*d*]pyrimidine moieties have a planar conformation (r.m.s. deviations = 0.020 and 0.012 Å). In the crystal, mol­ecules are linked through N—H⋯N, N—H⋯C and C—H⋯π non-covalent contacts into chains along the *c* axis, while neighbouring chains are connected *via* C—H⋯N inter­actions.

## Related literature

For the synthesis of 4-benzyl­amino­thieno[3,2-*d*]pyrimidine hydro­chloride, its NMR characterization (DMSO-*d_6_* solution) and biological activity, see: Crespo *et al.* (1998 ▶).
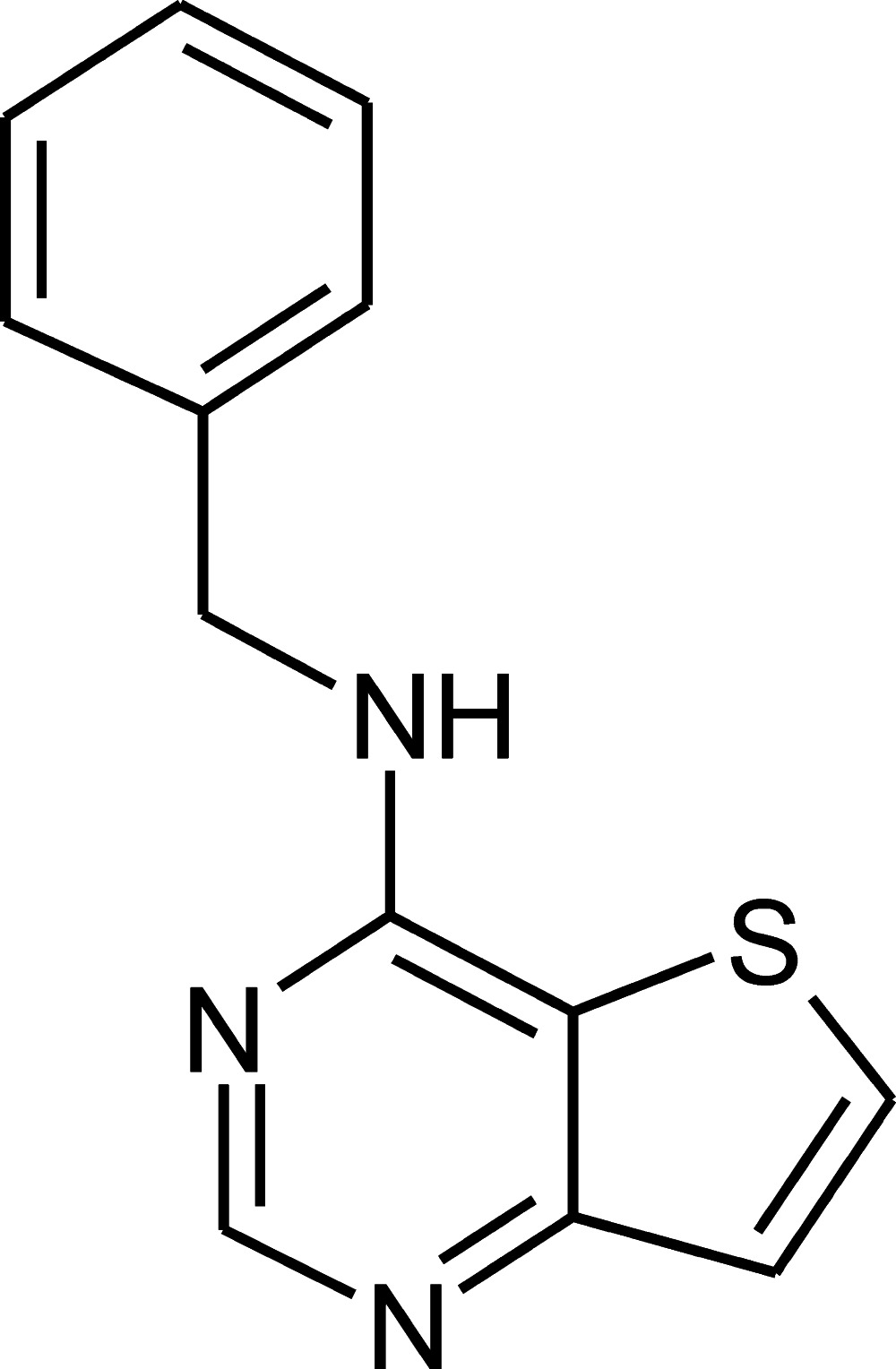


## Experimental

### 

#### Crystal data

C_13_H_11_N_3_S
*M* *_r_* = 241.31
Monoclinic, 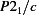

*a* = 19.3430 (4) Å
*b* = 9.46296 (16) Å
*c* = 12.8221 (2) Å
β = 94.3231 (17)°
*V* = 2340.30 (7) Å^3^
*Z* = 8
Mo *K*α radiation
μ = 0.26 mm^−1^
*T* = 120 K
0.40 × 0.40 × 0.25 mm

#### Data collection

Agilent Xcalibur Sapphire2 diffractometer
Absorption correction: multi-scan (*CrysAlis PRO*; Agilent, 2012 ▶) *T*_min_ = 0.905, *T*_max_ = 0.939
19406 measured reflections
4109 independent reflections
3528 reflections with *I* > 2σ(*I*)
*R*_int_ = 0.019

#### Refinement

*R*[*F* ^2^ > 2σ(*F* ^2^)] = 0.029
*wR*(*F* ^2^) = 0.078
*S* = 1.07
4109 reflections
307 parameters
H-atom parameters constrained
Δρ_max_ = 0.28 e Å^−3^
Δρ_min_ = −0.25 e Å^−3^

### 

Data collection: *CrysAlis PRO* (Agilent, 2012 ▶); cell refinement: *CrysAlis PRO*; data reduction: *CrysAlis PRO*; program(s) used to solve structure: *SHELXS97* (Sheldrick, 2008 ▶); program(s) used to refine structure: *SHELXL97* (Sheldrick, 2008 ▶); molecular graphics: *DIAMOND* (Brandenburg, 2011 ▶); software used to prepare material for publication: *publCIF* (Westrip, 2010 ▶).

## Supplementary Material

Crystal structure: contains datablock(s) I, global. DOI: 10.1107/S1600536813009537/tk5215sup1.cif

Structure factors: contains datablock(s) I. DOI: 10.1107/S1600536813009537/tk5215Isup2.hkl

Supplementary material file. DOI: 10.1107/S1600536813009537/tk5215Isup3.cml

Additional supplementary materials:  crystallographic information; 3D view; checkCIF report

## Figures and Tables

**Table 1:** Hydrogen-bond geometry (Å, °) *Cg* is the centroid of the C10–C15 ring.

*D*—H⋯*A*	*D*—H	H⋯*A*	*D*⋯*A*	*D*—H⋯*A*
N4—H4⋯N1^i^	0.88	2.13	2.999 (2)	167
N4*A*—H4*A*⋯N1*A* ^ii^	0.88	2.05	2.872 (2)	156
C7—H7⋯*Cg* ^iii^	0.95	2.58	3.5317 (13)	175
C2*A*—H2*A*⋯N3^iv^	0.95	2.67	3.527 (2)	150
C15—H15⋯N3*A*	0.95	2.58	3.4842 (18)	159

Symmetry codes: (i) 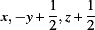; (ii) 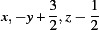; (iii) 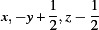; (iv) 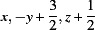.

**Fig. 1.**
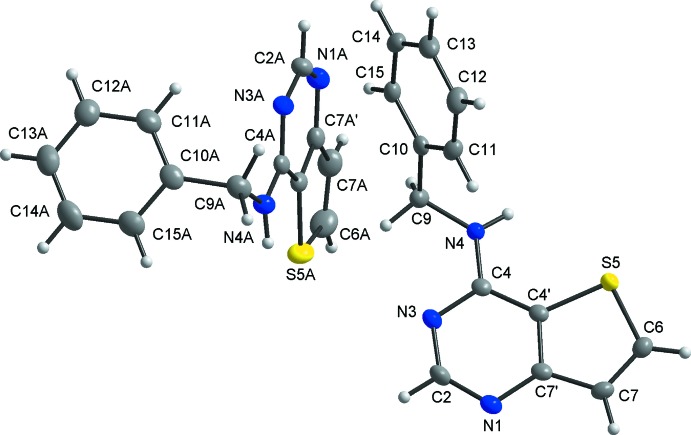
Two crystallographically independent molecules of the title compound with the non-hydrogen atoms depicted with anisotropic displacement ellipsoids at the 50% probability level and given with the atom numbering scheme.

**Fig. 2.**
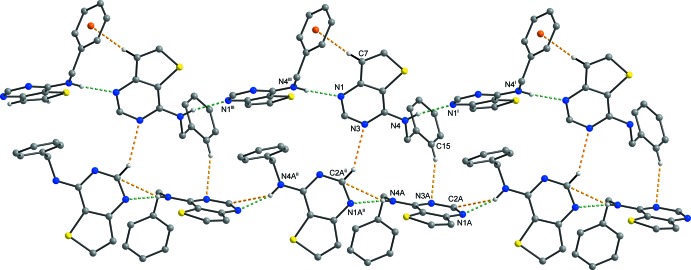
Part of the crystal structure, showing the formation of one-dimensional chains as well as non-covalent ineteractions within (N4—H4···N1 hydrogen bond (dashed green lines) and C7—H7···π, N4A—H4A···C2A (dashed orange lines)) and between (C15–H15···N3A and C2A—H2A···N3; dashed orange lines; see Table 1 for parameters) the chains.

## supplementary crystallographic information

### Comment

The essentially planar thieno[3,2-*d*]pyrimidine moiety is formed by six-membered (pyrimidine) and five-membered (thiophene) rings, which form a dihedral angle of 2.53 (4)° and 1.24 (4)° (for the molecule with the N1 and N1A atoms, respectively). The thieno[3,2-*d*]pyrimidine moiety is substituted by benzylamine at the C4 position (Fig. 1). The dihedral angles formed by the benzene and thieno[3,2-*d*]pyrimidine rings is 69.49 (4)° for the N1-molecule, and 79.05 (3)° for the N1A-molecule. The N4—H4···N1^i^ (for the molecule with the N1 atom) and N4A—H4A···N1A^ii^ (for the molecule with the N1A atom) hydrogen bonds together with other non-covalent contacts of the type N—H···C and C—H···π (Fig. 2, Table 1) connect the individual molecules into chains along the *c* axis (symmetry codes: i) *x*, –y+0.5, *z*+0.5; ii) *x*, –y+1.5, *z*–0.5). The neighbouring chains are connected through the C15–H15···N3A and and C2A—H2A···N3 interactions.

### Experimental

4-Chlorothieno[3,2-*d*]pyrimidine (10.0 mmol) was dissolved in 50 ml of 2-propanol. Benzylamine (13.0 mmol) and triethylamine (16.0 mmol) were subsequently added to the reaction mixture, which was stirred at 60 °C. The TLC control showed one spot after 18 h. After that, the mixture was evaporated and suspended in distilled water (20 ml). The product was collected by filtration, washed with distilled water and 2-propanol and dried in a desiccator over silica gel. Part of the product was recrystallized from acetone, which led to a crystalline product containing the crystals suitable for a single-crystal X-ray analysis. ^1^H NMR (DMF-*d_7_*, TMS, 298 K, p.p.m.): *δ* 8.52 (s, 1H, HC^2^), 8.41 (t, *J* = 6.5 Hz, 1H, HN^4^), 8.14 (d, *J* = 5.3 Hz, 1H, HC^6^), 7.44 (d, *J* = 7.7 Hz, 2H, HC^11,15^), 7.42 (d, *J* = 5.5 Hz, 1H, HC^7^), 7.33 (t, *J* = 7.7 Hz, 2H, HC^12,14^), 7.25 (t, *J* = 7.5 Hz, 1H, HC^13^), 4.87 (d, *J* = 6.0 Hz, 2H, HC^9^). ^13^C NMR (DMF-*d_7_*, TMS, 298 K, p.p.m.): *δ* 160.6 (C^7`^), 158.0 (C^4^), 155.4 (C^2^), 140.6 (C^10^), 133.2 (C^6^), 129.0 (C^12,14^), 128.2 (C^11,15^), 127.5 (C^13^), 125.4 (C^7^), 115.8 (C^4`^), 44.5 (C^9^). Analysis calculated for C_13_H_11_N_3_S_1_: C 64.7, H 4.6, N 17.4, S 13.3%; found: C 64.3, H 4.6, N 17.3, S 12.8%. Elemental analysis (C, H, N) was performed on a Thermo Scientific Flash 2000 CHNO-S Analyzer. The ^1^H and ^13^C NMR spectra (DMF-*d_7_* solutions, calibrated against tetramethylsilane) were collected at 298 K on a Varian 400 spectrometer at 400.00 and 100.58 MHz, respectively.

### Refinement

Non-hydrogen atoms were refined anisotropically and hydrogen atoms were located in difference maps and refined using the riding model with C—H = 0.95 (CH), C—H = 0.99 (CH_2_) Å, and N—H = 0.88 Å, with *U*_iso_(H) = 1.2*U*_eq_(CH, CH_2_, NH).

### Crystal data

**Table:**

C_13_H_11_N_3_S	*F*(000) = 1008
*M**_r_* = 241.31	*D*_x_ = 1.370 Mg m^−^^3^
Monoclinic, *P*2_1_/*c*	Mo *K*α radiation, λ = 0.71073 Å
Hall symbol: -P 2ybc	Cell parameters from 15514 reflections
*a* = 19.3430 (4) Å	θ = 3.0–33.2°
*b* = 9.46296 (16) Å	µ = 0.26 mm^−^^1^
*c* = 12.8221 (2) Å	*T* = 120 K
β = 94.3231 (17)°	Prism, colourless
*V* = 2340.30 (7) Å^3^	0.40 × 0.40 × 0.25 mm
*Z* = 8	

### Data collection

**Table:**

Agilent Xcalibur Sapphire2 diffractometer	4109 independent reflections
Radiation source: Enhance (Mo) X-ray Source	3528 reflections with *I* > 2σ(*I*)
Graphite monochromator	*R*_int_ = 0.019
Detector resolution: 8.3611 pixels mm^-1^	θ_max_ = 25.0°, θ_min_ = 3.0°
ω scans	*h* = −23→23
Absorption correction: multi-scan (*CrysAlis PRO*; Agilent, 2012)	*k* = −11→11
*T*_min_ = 0.905, *T*_max_ = 0.939	*l* = −15→15
19406 measured reflections	

### Refinement

**Table:**

Refinement on *F*^2^	Primary atom site location: structure-invariant direct methods
Least-squares matrix: full	Secondary atom site location: difference Fourier map
*R*[*F*^2^ > 2σ(*F*^2^)] = 0.029	Hydrogen site location: inferred from neighbouring sites
*wR*(*F*^2^) = 0.078	H-atom parameters constrained
*S* = 1.07	*w* = 1/[σ^2^(*F*_o_^2^) + (0.0476*P*)^2^ + 0.4186*P*] where *P* = (*F*_o_^2^ + 2*F*_c_^2^)/3
4109 reflections	(Δ/σ)_max_ < 0.001
307 parameters	Δρ_max_ = 0.28 e Å^−^^3^
0 restraints	Δρ_min_ = −0.25 e Å^−^^3^

### Special details

**Table:**

Geometry. All e.s.d.'s (except the e.s.d. in the dihedral angle between two l.s. planes) are estimated using the full covariance matrix. The cell e.s.d.'s are taken into account individually in the estimation of e.s.d.'s in distances, angles and torsion angles; correlations between e.s.d.'s in cell parameters are only used when they are defined by crystal symmetry. An approximate (isotropic) treatment of cell e.s.d.'s is used for estimating e.s.d.'s involving l.s. planes.
Refinement. Refinement of *F*^2^ against ALL reflections. The weighted *R*-factor *wR* and goodness of fit *S* are based on *F*^2^, conventional *R*-factors *R* are based on *F*, with *F* set to zero for negative *F*^2^. The threshold expression of *F*^2^ > σ(*F*^2^) is used only for calculating *R*-factors(gt) *etc*. and is not relevant to the choice of reflections for refinement. *R*-factors based on *F*^2^ are statistically about twice as large as those based on *F*, and *R*- factors based on ALL data will be even larger.

### Fractional atomic coordinates and isotropic or equivalent isotropic displacement parameters (Å^2^)

**Table:**

	*x*	*y*	*z*	*U*_iso_*/*U*_eq_	
S5	0.108822 (18)	0.10694 (4)	0.60102 (3)	0.02112 (11)	
S5A	0.45872 (2)	0.62119 (4)	0.66155 (3)	0.03086 (12)	
N3A	0.31415 (6)	0.88825 (12)	0.75775 (9)	0.0224 (3)	
N3	0.19707 (6)	0.41535 (12)	0.44021 (8)	0.0206 (3)	
C2A	0.33340 (8)	0.87296 (15)	0.85980 (11)	0.0248 (3)	
H2A	0.3066	0.9241	0.9062	0.030*	
C2	0.19194 (7)	0.34543 (15)	0.34913 (10)	0.0223 (3)	
H2	0.2092	0.3942	0.2917	0.027*	
N1A	0.38455 (6)	0.79651 (13)	0.90461 (9)	0.0271 (3)	
N1	0.16633 (6)	0.21820 (12)	0.32771 (8)	0.0222 (3)	
C7A'	0.42113 (7)	0.72184 (14)	0.83595 (11)	0.0227 (3)	
C7'	0.14191 (7)	0.15142 (14)	0.41194 (10)	0.0186 (3)	
C4A'	0.40431 (7)	0.72635 (14)	0.72891 (11)	0.0204 (3)	
C4'	0.14582 (7)	0.21307 (14)	0.51077 (10)	0.0174 (3)	
N4	0.18145 (6)	0.41308 (12)	0.61780 (8)	0.0198 (3)	
H4	0.1750	0.3622	0.6737	0.024*	
N4A	0.33088 (6)	0.82477 (13)	0.58732 (9)	0.0238 (3)	
H4A	0.3504	0.7673	0.5443	0.029*	
C4	0.17530 (6)	0.34889 (14)	0.52409 (10)	0.0170 (3)	
C4A	0.34922 (7)	0.81394 (14)	0.68957 (10)	0.0193 (3)	
C7A	0.47914 (8)	0.63230 (16)	0.86262 (13)	0.0323 (4)	
H7A	0.4982	0.6165	0.9321	0.039*	
C7	0.10891 (7)	0.01643 (15)	0.41142 (10)	0.0221 (3)	
H7	0.1017	−0.0415	0.3511	0.026*	
C6A	0.50344 (8)	0.57307 (18)	0.77755 (13)	0.0373 (4)	
H6A	0.5419	0.5103	0.7809	0.045*	
C6	0.08920 (7)	−0.01926 (15)	0.50635 (11)	0.0232 (3)	
H6	0.0666	−0.1058	0.5199	0.028*	
C9	0.19815 (7)	0.56162 (15)	0.63204 (11)	0.0232 (3)	
H9A	0.2132	0.6007	0.5658	0.028*	
H9B	0.2371	0.5716	0.6862	0.028*	
C9A	0.28012 (7)	0.92695 (16)	0.54290 (11)	0.0275 (3)	
H9D	0.2434	0.9403	0.5917	0.033*	
H9C	0.2581	0.8894	0.4764	0.033*	
C10A	0.31332 (7)	1.06765 (16)	0.52284 (11)	0.0264 (3)	
C10	0.13638 (7)	0.64490 (14)	0.66460 (10)	0.0194 (3)	
C11	0.06933 (7)	0.60827 (14)	0.62788 (11)	0.0227 (3)	
H11	0.0622	0.5333	0.5790	0.027*	
C11A	0.32231 (7)	1.16842 (17)	0.60148 (12)	0.0297 (3)	
H11A	0.3050	1.1508	0.6676	0.036*	
C12A	0.35621 (8)	1.29412 (17)	0.58469 (13)	0.0344 (4)	
H12A	0.3618	1.3624	0.6390	0.041*	
C12	0.01270 (8)	0.68002 (15)	0.66191 (12)	0.0285 (3)	
H12	−0.0329	0.6538	0.6364	0.034*	
C13	0.02237 (8)	0.78964 (15)	0.73294 (12)	0.0291 (3)	
H13	−0.0164	0.8380	0.7570	0.035*	
C13A	0.38197 (8)	1.32031 (18)	0.48879 (13)	0.0360 (4)	
H13A	0.4061	1.4057	0.4776	0.043*	
C14	0.08889 (8)	0.82823 (15)	0.76859 (11)	0.0265 (3)	
H14	0.0959	0.9042	0.8167	0.032*	
C14A	0.37252 (9)	1.22257 (19)	0.40976 (13)	0.0388 (4)	
H14A	0.3895	1.2412	0.3435	0.047*	
C15A	0.33837 (8)	1.09711 (18)	0.42644 (12)	0.0328 (4)	
H15A	0.3320	1.0302	0.3713	0.039*	
C15	0.14548 (7)	0.75657 (14)	0.73447 (11)	0.0228 (3)	
H15	0.1910	0.7842	0.7592	0.027*	

### Atomic displacement parameters (Å^2^)

**Table:**

	*U*^11^	*U*^22^	*U*^33^	*U*^12^	*U*^13^	*U*^23^
S5	0.0280 (2)	0.01963 (19)	0.01620 (18)	−0.00222 (13)	0.00445 (14)	0.00056 (13)
S5A	0.0272 (2)	0.0318 (2)	0.0339 (2)	0.00627 (15)	0.00492 (16)	−0.00622 (16)
N3A	0.0211 (6)	0.0247 (6)	0.0218 (6)	0.0000 (5)	0.0039 (5)	−0.0010 (5)
N3	0.0206 (6)	0.0243 (6)	0.0169 (6)	−0.0002 (5)	0.0024 (5)	0.0031 (5)
C2A	0.0263 (8)	0.0272 (8)	0.0217 (7)	−0.0011 (6)	0.0061 (6)	−0.0019 (6)
C2	0.0221 (7)	0.0294 (8)	0.0158 (7)	0.0000 (6)	0.0033 (5)	0.0041 (6)
N1A	0.0291 (7)	0.0310 (7)	0.0212 (6)	−0.0007 (5)	0.0017 (5)	0.0004 (5)
N1	0.0233 (6)	0.0271 (7)	0.0163 (6)	0.0019 (5)	0.0027 (5)	0.0012 (5)
C7A'	0.0219 (7)	0.0210 (7)	0.0252 (7)	−0.0047 (5)	0.0004 (6)	0.0020 (6)
C7'	0.0168 (7)	0.0225 (7)	0.0165 (7)	0.0055 (5)	0.0012 (5)	0.0008 (5)
C4A'	0.0185 (7)	0.0186 (7)	0.0244 (7)	−0.0039 (5)	0.0046 (5)	−0.0011 (6)
C4'	0.0162 (7)	0.0198 (7)	0.0163 (6)	0.0048 (5)	0.0018 (5)	0.0018 (5)
N4	0.0260 (6)	0.0182 (6)	0.0155 (6)	−0.0013 (5)	0.0037 (5)	0.0004 (5)
N4A	0.0262 (6)	0.0262 (7)	0.0191 (6)	0.0014 (5)	0.0020 (5)	−0.0008 (5)
C4	0.0137 (6)	0.0205 (7)	0.0168 (7)	0.0047 (5)	0.0010 (5)	0.0014 (5)
C4A	0.0176 (7)	0.0198 (7)	0.0206 (7)	−0.0062 (5)	0.0029 (5)	0.0000 (6)
C7A	0.0294 (8)	0.0327 (9)	0.0334 (9)	0.0006 (7)	−0.0069 (7)	0.0046 (7)
C7	0.0253 (7)	0.0220 (7)	0.0186 (7)	0.0034 (6)	−0.0009 (6)	−0.0042 (6)
C6A	0.0272 (9)	0.0331 (9)	0.0505 (11)	0.0095 (7)	−0.0028 (7)	0.0015 (8)
C6	0.0257 (8)	0.0191 (7)	0.0247 (7)	−0.0002 (6)	0.0003 (6)	−0.0014 (6)
C9	0.0240 (8)	0.0222 (7)	0.0239 (7)	−0.0042 (6)	0.0050 (6)	−0.0033 (6)
C9A	0.0221 (8)	0.0361 (9)	0.0237 (7)	0.0012 (6)	−0.0027 (6)	0.0028 (6)
C10A	0.0176 (7)	0.0332 (8)	0.0280 (8)	0.0066 (6)	−0.0013 (6)	0.0080 (7)
C10	0.0238 (7)	0.0174 (7)	0.0173 (7)	−0.0019 (5)	0.0033 (5)	0.0040 (5)
C11	0.0281 (8)	0.0181 (7)	0.0213 (7)	−0.0005 (6)	−0.0015 (6)	0.0008 (6)
C11A	0.0234 (8)	0.0353 (9)	0.0307 (8)	0.0056 (6)	0.0036 (6)	0.0045 (7)
C12A	0.0296 (9)	0.0320 (9)	0.0413 (10)	0.0048 (7)	0.0009 (7)	0.0031 (7)
C12	0.0239 (8)	0.0245 (8)	0.0367 (9)	0.0009 (6)	−0.0007 (6)	0.0057 (7)
C13	0.0315 (9)	0.0209 (8)	0.0360 (8)	0.0064 (6)	0.0105 (7)	0.0058 (6)
C13A	0.0299 (9)	0.0316 (9)	0.0465 (10)	0.0038 (7)	0.0026 (7)	0.0133 (8)
C14	0.0398 (9)	0.0161 (7)	0.0245 (8)	−0.0010 (6)	0.0078 (6)	−0.0002 (6)
C14A	0.0365 (9)	0.0459 (10)	0.0344 (9)	0.0039 (8)	0.0050 (7)	0.0172 (8)
C15A	0.0330 (9)	0.0384 (9)	0.0266 (8)	0.0055 (7)	0.0000 (7)	0.0067 (7)
C15	0.0275 (8)	0.0190 (7)	0.0218 (7)	−0.0055 (6)	0.0025 (6)	0.0016 (6)

### Geometric parameters (Å, º)

**Table:**

S5—C6	1.7246 (14)	C6A—H6A	0.9500
S5—C4'	1.7277 (13)	C6—H6	0.9500
S5A—C6A	1.7245 (17)	C9—C10	1.5159 (19)
S5A—C4A'	1.7266 (14)	C9—H9A	0.9900
N3A—C2A	1.3410 (19)	C9—H9B	0.9900
N3A—C4A	1.3447 (18)	C9A—C10A	1.509 (2)
N3—C2	1.3395 (18)	C9A—H9D	0.9900
N3—C4	1.3408 (17)	C9A—H9C	0.9900
C2A—N1A	1.3216 (19)	C10A—C11A	1.389 (2)
C2A—H2A	0.9500	C10A—C15A	1.389 (2)
C2—N1	1.3228 (19)	C10—C15	1.3880 (19)
C2—H2	0.9500	C10—C11	1.390 (2)
N1A—C7A'	1.3671 (19)	C11—C12	1.387 (2)
N1—C7'	1.3660 (17)	C11—H11	0.9500
C7A'—C4A'	1.387 (2)	C11A—C12A	1.383 (2)
C7A'—C7A	1.427 (2)	C11A—H11A	0.9500
C7'—C4'	1.3918 (18)	C12A—C13A	1.383 (2)
C7'—C7	1.428 (2)	C12A—H12A	0.9500
C4A'—C4A	1.4127 (19)	C12—C13	1.384 (2)
C4'—C4	1.4112 (19)	C12—H12	0.9500
N4—C4	1.3436 (17)	C13—C14	1.382 (2)
N4—C9	1.4507 (18)	C13—H13	0.9500
N4—H4	0.8800	C13A—C14A	1.374 (2)
N4A—C4A	1.3361 (17)	C13A—H13A	0.9500
N4A—C9A	1.4626 (18)	C14—C15	1.386 (2)
N4A—H4A	0.8800	C14—H14	0.9500
C7A—C6A	1.342 (2)	C14A—C15A	1.383 (2)
C7A—H7A	0.9500	C14A—H14A	0.9500
C7—C6	1.3457 (19)	C15A—H15A	0.9500
C7—H7	0.9500	C15—H15	0.9500
			
C6—S5—C4'	90.65 (6)	N4—C9—C10	111.45 (11)
C6A—S5A—C4A'	90.33 (7)	N4—C9—H9A	109.3
C2A—N3A—C4A	117.47 (12)	C10—C9—H9A	109.3
C2—N3—C4	117.42 (12)	N4—C9—H9B	109.3
N1A—C2A—N3A	128.83 (13)	C10—C9—H9B	109.3
N1A—C2A—H2A	115.6	H9A—C9—H9B	108.0
N3A—C2A—H2A	115.6	N4A—C9A—C10A	111.58 (11)
N1—C2—N3	129.24 (13)	N4A—C9A—H9D	109.3
N1—C2—H2	115.4	C10A—C9A—H9D	109.3
N3—C2—H2	115.4	N4A—C9A—H9C	109.3
C2A—N1A—C7A'	114.19 (12)	C10A—C9A—H9C	109.3
C2—N1—C7'	113.79 (11)	H9D—C9A—H9C	108.0
N1A—C7A'—C4A'	121.81 (13)	C11A—C10A—C15A	118.36 (15)
N1A—C7A'—C7A	126.04 (13)	C11A—C10A—C9A	120.90 (13)
C4A'—C7A'—C7A	112.15 (13)	C15A—C10A—C9A	120.68 (14)
N1—C7'—C4'	121.84 (13)	C15—C10—C11	118.57 (13)
N1—C7'—C7	126.25 (12)	C15—C10—C9	120.62 (12)
C4'—C7'—C7	111.89 (12)	C11—C10—C9	120.78 (12)
C7A'—C4A'—C4A	119.06 (12)	C12—C11—C10	120.68 (13)
C7A'—C4A'—S5A	111.77 (11)	C12—C11—H11	119.7
C4A—C4A'—S5A	129.15 (11)	C10—C11—H11	119.7
C7'—C4'—C4	119.06 (12)	C12A—C11A—C10A	120.81 (14)
C7'—C4'—S5	111.67 (10)	C12A—C11A—H11A	119.6
C4—C4'—S5	129.22 (10)	C10A—C11A—H11A	119.6
C4—N4—C9	123.67 (11)	C11A—C12A—C13A	119.95 (16)
C4—N4—H4	118.2	C11A—C12A—H12A	120.0
C9—N4—H4	118.2	C13A—C12A—H12A	120.0
C4A—N4A—C9A	123.62 (12)	C13—C12—C11	120.22 (14)
C4A—N4A—H4A	118.2	C13—C12—H12	119.9
C9A—N4A—H4A	118.2	C11—C12—H12	119.9
N3—C4—N4	119.55 (12)	C14—C13—C12	119.49 (14)
N3—C4—C4'	118.59 (12)	C14—C13—H13	120.3
N4—C4—C4'	121.85 (12)	C12—C13—H13	120.3
N4A—C4A—N3A	119.21 (12)	C14A—C13A—C12A	119.88 (16)
N4A—C4A—C4A'	122.19 (12)	C14A—C13A—H13A	120.1
N3A—C4A—C4A'	118.60 (12)	C12A—C13A—H13A	120.1
C6A—C7A—C7A'	111.74 (14)	C13—C14—C15	120.26 (14)
C6A—C7A—H7A	124.1	C13—C14—H14	119.9
C7A'—C7A—H7A	124.1	C15—C14—H14	119.9
C6—C7—C7'	112.17 (12)	C13A—C14A—C15A	120.15 (16)
C6—C7—H7	123.9	C13A—C14A—H14A	119.9
C7'—C7—H7	123.9	C15A—C14A—H14A	119.9
C7A—C6A—S5A	114.02 (12)	C14A—C15A—C10A	120.83 (16)
C7A—C6A—H6A	123.0	C14A—C15A—H15A	119.6
S5A—C6A—H6A	123.0	C10A—C15A—H15A	119.6
C7—C6—S5	113.61 (11)	C14—C15—C10	120.76 (13)
C7—C6—H6	123.2	C14—C15—H15	119.6
S5—C6—H6	123.2	C10—C15—H15	119.6

### Hydrogen-bond geometry (Å, º)

Cg is the centroid of the C10–C15 ring.

**Table:**

*D*—H···*A*	*D*—H	H···*A*	*D*···*A*	*D*—H···*A*
N4—H4···N1^i^	0.88	2.13	2.999 (2)	167
N4*A*—H4*A*···N1*A*^ii^	0.88	2.05	2.872 (2)	156
C7—H7···*Cg*^iii^	0.95	2.58	3.5317 (13)	175
C2*A*—H2*A*···N3^iv^	0.95	2.67	3.527 (2)	150
C15—H15···N3*A*	0.95	2.58	3.4842 (18)	159

Symmetry codes: (i) *x*, −*y*+1/2, *z*+1/2; (ii) *x*, −*y*+3/2, *z*−1/2; (iii) *x*, −*y*+1/2, *z*−1/2; (iv) *x*, −*y*+3/2, *z*+1/2.
